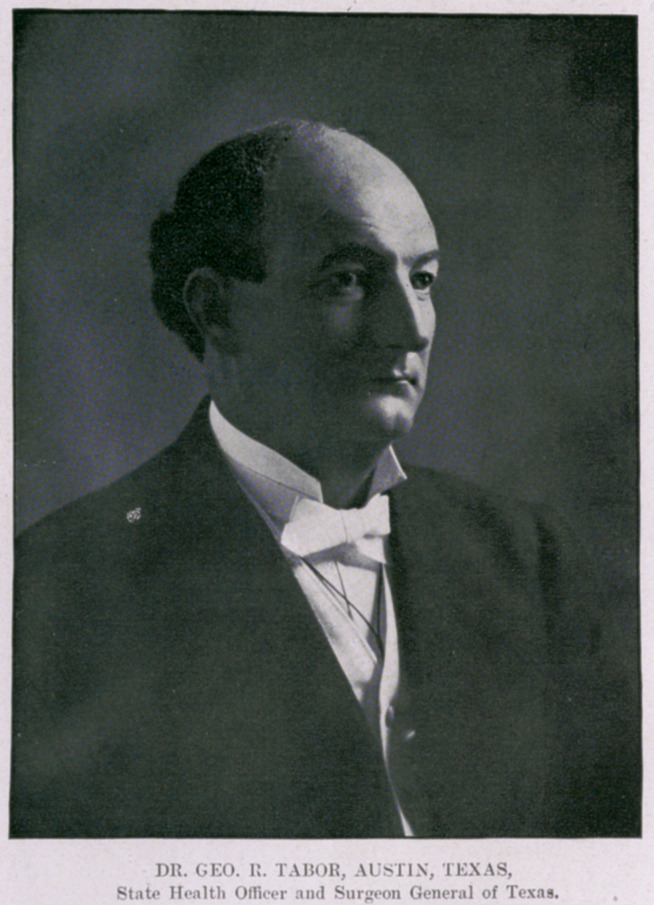# George R. Tabor, M. D., State Health Officer and Surgeon General of Texas

**Published:** 1906-07

**Authors:** 


State Health Officer and Surgeon General of Texas.

THE

### TEXAS MEDICAL JOURNAL.

Established July, 1885.

F. E. DANIEL, M. D., - - - - - Editor, Publisher and Proprietor.

PUBLISHED MONTHLY.—SUBSCRIPTION $1.00 A YEAR.

VOL. XXII. AUSTIN, JULY, 1906. No. 1.

The publisher is not responsible for the views of contributors.

### George R. Tabor, M. D., State Health Officer and Surgeon General of Texas.

BY THE EDITOR.

Dr. Tabor was born in Caldwell county, Texas, August 30, 1864. His parents moved to Bryan in 1867, where he was reared. He was educated in private and public schools and the Agricultural and Mechanical College. He graduated in medicine at Louisville Medical College in 1888, and held the honor of being president of the graduating class. After graduating he returned to Bryan and practiced medicine, and was appointed County Health Officer of Brazos county, and City Health Officer of Bryan for a number of years, during which time he encountered several epidemics of smallpox and some yellow fever. Prior to his appointment as State Health Officer he made an enviable record as a health officer in Central Texas, and made a special study of contagious diseases, in which he spent four or more months in New Orleans during one summer for that purpose; therefore, he came into the office well equipped for the work ahead of him. During his stay in New Orleans he was acting as special inspector for the State of Texas, having been appointed to that position by Governor Sayers, who afterwards appointed Dr. Tabor State Health Officer, which was a few days after his return from New Orleans, to succeed Dr. Blunt, who resigned from office.

Yellow fever was introduced into Laredo and San Antonio from Mexico, but he succeeded in confining it to certain limits, preventing its spreading throughout the State. After the epidemics at Laredo and San Antonio he conducted a systematic sanitary campaign right up to the time of the New Orleans epidemic.

Dr. Tabor originated the uniform system of quarantine between the Gulf States. When he went into office there was no uniform system of quarantine, but through his efforts the regulations at Mobile, New Orleans, and Texas ports are now uniform. The first conference of the States of Alabama, Louisiana, and Texas was instigated and called by him and the representatives of the Health Departments of the States of Louisiana and Alabama met with him in Galveston, where they formulated the regulations, which are now in effect, governing the inspection and disinfection of vessels arriving at all of these ports, which places all Southern ports on the same basis. The representatives of the States of Alabama, Louisiana, and Texas meet once each year to discuss these regulations, alternately at Galveston, New Orleans and Mobile. Previous to these regulations there were constant bickerings at the several Gulf ports as to the advantages of one and the disadvantages of the other, resulting, of course, in a lack of harmony in the quarantine service, and, primarily, lack of protection to the people, but now each port is equal to the other in matters of shipping and commerce. This was a victory for Galveston, for Texas and her State Health Officer had enforced safe quarantine while the other ports had been quite lax in order to attract commerce. When uniformity came Galveston and other Texas ports went on the same footing with New Orleans, Mobile and other Gulf ports. Several conferences have been held since the first, in which the rules have been changed to meet the new conditions.

The second achievement of Dr. Tabor was his successful campaign with the Mexican health authorities for a cleaner Mexico. He treated with the Mexican authorities at length, visiting their country at the head of a commission of the Southern States health officials, which was organized by Dr. Tabor, and was treated with great courtesy and consideration by Dr. Eduardo Liceaga, President of the Superior Board of Health of Mexico, and his brother Mexican health officials. They visited a number of towns, including some of the formerly infected districts, and made numerous recommendations as to what should be done. Most of these suggestions were adopted, which resulted in modern sanitary methods being adopted and the complete eradication of yellow fever at Mexican places and near Texas, thereby removing a serious and constant danger to the health of this State, and all other Southern States through Texas.

At the time of the bubonic plague infection in San Francisco, and when the country was stirred with the prospect of its becoming epidemic in the United States, Dr. Tabor was the first State Health Officer to suggest to Surgeon-General Wyman, of the United States Public Health and Marine Hospital Service, to visit San Francisco and look into the matter personally. Dr. Tabor was joined in this by Dr. Souchon, late President of the Louisiana State Board of Health, and together they went to Washington to urge upon Surgeon-General Wyman to look into the matter personally. The Surgeon-General, with Dr. Tabor, visited San Francisco and made a thorough investigation, and Dr. Tabor told the California authorities that he had implicit faith in Dr. Gardner, and that any report Dr. Gardner might make of the bubonic plague situation would be accepted as exact, and all the other States would abide with it. It was not long after this that the Governor of California did appoint Dr. Gardner as President of the State Board of Health of California, and there was immediate harmony in quarantine regulations at the Texas border, against which California merchants and importers at San Francisco had complained bitterly. Thus Dr. Tabor's visit had much to do with the satisfactory adjustment of the bubonic plague quarantine situation.

Dr. Tabor's conduct in the last trying and successful campaign to keep yellow fever out of Texas made him a National reputation. His administration has been characterized by intelligence, vigor and zeal, and has been effective and eminently satisfactory. He combines, with a marked administrative and executive ability, the rare faculty of reconciling conflicting interests and opinions, and of making friends and supporters of both sides. In fact, he is the right man in the right place. The people of Galveston, as a mark of appproval and appreciation, gave him a large honorarium, and a trip to Europe for himself and his charming and accomplished bride, *nee* Miss Ann Barton, of Austin.

## Figures and Tables

**Figure f1:**